# Ultrasound-Guided Percutaneous Microwave Ablation for Solid Benign Thyroid Nodules: Comparison of MWA versus Control Group

**DOI:** 10.1155/2017/9724090

**Published:** 2017-11-23

**Authors:** Wenjun Wu, Xiaohua Gong, Qi Zhou, Xiong Chen, Xiaojun Chen

**Affiliations:** Department of Endocrinology, The First Affiliated Hospital of Wenzhou Medical University, Ouhai District, Wenzhou, China

## Abstract

**Background:**

The aim of this research is to investigate the feasibility of percutaneous ultrasound-guided microwave ablation (MWA) for benign solid thyroid nodules.

**Methods:**

Ultrasound-guided percutaneous microwave ablation was performed for 90 benign solid thyroid nodules in 75 patients. The volume changes of the nodules were evaluated before and after microwave ablation, and the cosmetic grading and clinical symptoms were assessed as well.

**Results:**

The volume of all the 90 benign thyroid nodules obviously decreased after microwave ablation at 3-, 6-, 9-, and 12-month follow-ups (*p* < 0.01), while that of the control group increased at the follow-up of 12 months (*p* < 0.01). The volume reduction rate (VRR) at 3-, 6-, 9-, and 12-month follow-ups was 55.98%, 69.31%, 76.65%, and 84.67% in the MWA group, respectively. The cosmetic problems and clinical symptoms were also improved in the MWA group. All the patients are well tolerated to the procedure. Hoarseness occurred in 2 cases (2.7%) and Horner syndrome in 1 case (1.3%), and 1 patient (1.3%) developed slight burn on cervical skin.

**Conclusions:**

Ultrasound-guided percutaneous microwave ablation is a practical method for treating benign solid thyroid nodules, and the complications were acceptable. The trial is registered with clinicaltrials.gov with the registration number NCT03057925.

## 1. Introduction

Thyroid nodules (TNs) are a common clinical disease, discovered by palpation in 3 to 7% and by ultrasound in about 50% of the general population [1]. Recent epidemiological studies indicated that the prevalence of thyroid nodules in the general population in Chinese men and women was 24.1% and 34.7%, respectively [2]. For the residents over 40 years old, thyroid nodule incidence is up to 46.6% [3]. Prevalence increased rapidly promotes the greater demand for more tailored treatment. However, there is in some dilemma for the doctors. For benign thyroid nodules, the medical conservative treatment such as levothyroxine suppressive therapy will not make it disappear, and the patients will be exposed to more risk if they received surgery, such as iatrogenic hypothyroidism, recurrent laryngeal nerve palsy, hypoparathyroidism, and other complications [4]. Many patients are with great anxiety. More tailored and minimal invasive modalities are needed. The last decades have witnessed the development of several nonsurgical, image-guided, minimally invasive approaches for treatment of TNs. The applications of thermal ablation to treat thyroid benign nodules showed obvious advantages [5–10]. Although there have been several studies comparing MWA with other methods or comparing devices [11, 12], to our knowledge, there has been no report of a comparison of the efficacy of MWA and control group. The aim of our study was to define the effectiveness of MWA in reducing nodule volume and relieving nodule-related clinical problems and to exclude an effect due to spontaneous nodule reduction by comparing treatment with control groups.

## 2. Material and Methods

### 2.1. Patients

A total 115 patients with 144 benign thyroid nodules were recruited from January to December 2015 in a thyroid center of one medical institution. The inclusion criteria for all the patients were as follows according to the current RFA guidelines [13]: (1) with solid or predominantly solid nodules on ultrasound (solid component > 75%); (2) with complaints of pressure symptoms, throat constraint, and/or swallowing difficulty or esthetic problems; (3) with benign cytology that means colloid and sheets of follicular cells without atypia, class 2 [14]; (4) without a history of treatment and refused to surgery; (5) and with normal thyroid function. The MWA group composed of those who received microwave ablation for the treatment. In the same period, patients who met the abovementioned inclusion criteria chose follow-up with periodical ultrasound checkup instead of any medical treatment made up of a control group. All the nodules had a benign diagnosis by ultrasound-guided fine needle aspiration cytology (FNAC). The MWA group consists of 64 females and 11 males, while the control group consists of 29 females and 11 males. All patients signed a written informed consent. The protocol was approved by the Ethics Committee of the First Affiliated Hospital of Wenzhou Medical University. The two groups in terms of age, gender, nodule locations, and thyroid hormones were compared, and the differences were not significant (Table 1).

### 2.2. Equipment and Preoperative Preparation

Nanjing YIGAO company ECO-100 multifunctional microwave therapeutic instrument was applied with disposable microwave ablation needle antenna (16 G). The antenna type (10 cm in total length, 1.6 mm in diameter, and 3 mm in length of active tip) is suitable for superficial organs. Output power setting was 35 W, with a frequency of 2450 MHz, and the internally cooled needle antenna with normal saline for cold circulation fluid was used. The diameters, composition, and vascularity of nodules were examined by Phillip iU22 color Doppler ultrasonic diagnostic apparatus, probe frequency 5~12 MHz. Preoperative, intraoperative, and postoperative of thyroid nodules were examined by a two-dimensional color Doppler flow, and contrast-enhanced ultrasonography (CEUS) examination was also performed.

Laboratory data including blood biochemistry analysis, complete blood count, blood coagulation test, and thyroid function (thyroid stimulating hormone, triiodothyronine, thyroxine, free triiodothyronine, and free thyroxine) were assayed, and electrocardiogram was examined. A multiparametric monitor was connected to the patient showing continuous blood pressure, oxyhemoglobin saturation, and electrocardiogram during the procedure.

### 2.3. Procedure

With the supine cervical extension, local anesthesia with 2% lidocaine was performed on the puncture site. After a small incision(<2 mm in length) was made, under the US guidance, a mixture of 2% lidocaine and 0.9% normal saline was infused into the surrounding thyroid capsule, a so-called “hydrodissection technique” to provide a safe barrier to prevent thermal damage to carotid artery and “danger triangle” where the trachea, esophagus, and recurrent laryngeal nerve are located [15]. Under the ultrasound guidance, the disposable microwave antenna was placed percutaneously into the nodule along its short axis, then pedal started microwave therapeutic instrument. During the microwave ablation, a power output of 35 W was usually used, and the variations in the echoes of the nodule were monitored by real-time ultrasound. The procedure was conducted in “moving-shot technique” [8]. The extent of ablation area was presumed by the echogenic change around the antenna. If the transient hyperechoic zone did not completely cover the entire nodule at one site, the tip of the antenna was moved backward. The microwave antenna was repositioned, and other parts of the nodule were treated when necessary. The ablation was not stopped until the transient hyperechoic zone covered the whole nodule. Before and after ablation, the application of high-frequency ultrasound on nodule location, size, texture, and with surrounding tissue adjacent relations was performed and recorded. Before and by the end of procedure, the nodules were examined by contrast-enhanced ultrasound observation of filling defect area. Nonenhancement was shown as a consequence of coagulative necrosis induced by MWA. Time consumed for each nodule was recorded, and mechanical compression for 30 minutes was needed on the site after the operation, so as to prevent bleeding or hematoma formation.

### 2.4. Assessment and Follow-Up of Preablation and Postablation

Before ablation and after that of 1, 3, 6, 9, and 12 months, respectively, the patients in both groups returned to our hospital for a follow-up review. Preoperative and postoperative thyroid function tests, thyroid globulin antibody, thyroid peroxidase antibody, and thyroid ultrasonography were performed. Changes in volume, echogenicity, and intranodular vascularity were all evaluated. Ultrasonography was performed by fixed technologists in the same ultrasound machine, and three orthogonal diameters of thyroid nodules were measured. The volume of the nodules was calculated by the following equation: *V* = *πabc*/6 (*V*: volume, *a*: the largest diameter; *b* and *c*: the other two perpendicular diameters).

The volume reduce rate (VRR) was assessed by a US imaging and was calculated by the following equation: volume reduction rate (%) = 100 × (initial volume − final volume)/initial volume. The complications during or after the ablation were also evaluated by the clinical signs and symptoms.

Clinical symptoms were evaluated using the symptom grading scores (visual analog scale, 0–10 cm), and the cosmetic grading scores (grade 1: no palpable mass; grade 2: invisible but palpable mass; grade 3: visible mass only by experienced clinician's eyes; and grade 4: easily visible mass) [16].

### 2.5. Statistical Analysis

Data analysis was performed with statistical software (SPSS for Windows version 19.0 SPSS IBM Corp., New York, NY). Quantitative data were expressed as mean ± standard deviation (SD), and *χ*^2^ test were used to compare sex and the number of nodule locations. Quantitative data between the two groups were compared by means of the Mann–Whitney *U* tests. The follow-up nodule volume and VRR of the nodule after MWA were compared with baseline volume by means of the Wilcoxon tests. *p* < 0.05 was considered statistically significant.

## 3. Results

### 3.1. Change of Thyroid Nodule Volume after Microwave Ablation

Ninety nodules were performed procedure in the MWA group for single-session treatment. The mean ablation time was 6.97 ± 5.13 minutes. The mean thyroid nodular volume of the MWA group decreased from 6.61 ± 4.65 ml (range, 1.11~22.6 ml) to 0.87 ± 0.99 ml (range, 0.05 ml~4.34 ml) (*p* < 0.001) at 1-year follow-up visit. Meanwhile, the mean volume in the control group increased from 5.34 ± 3.88 ml (range, 1.57~14.2 ml) to 6.50 ± 4.36 ml (range, 2.51~16.49 ml) (*p* < 0.001). When compared with the control group, the thyroid nodule volume in the MWA group was significantly reduced at 3-, 6-, 9-, and 12-month follow-up visits (*p* < 0.001) (Table 2).

**Figure 1:**
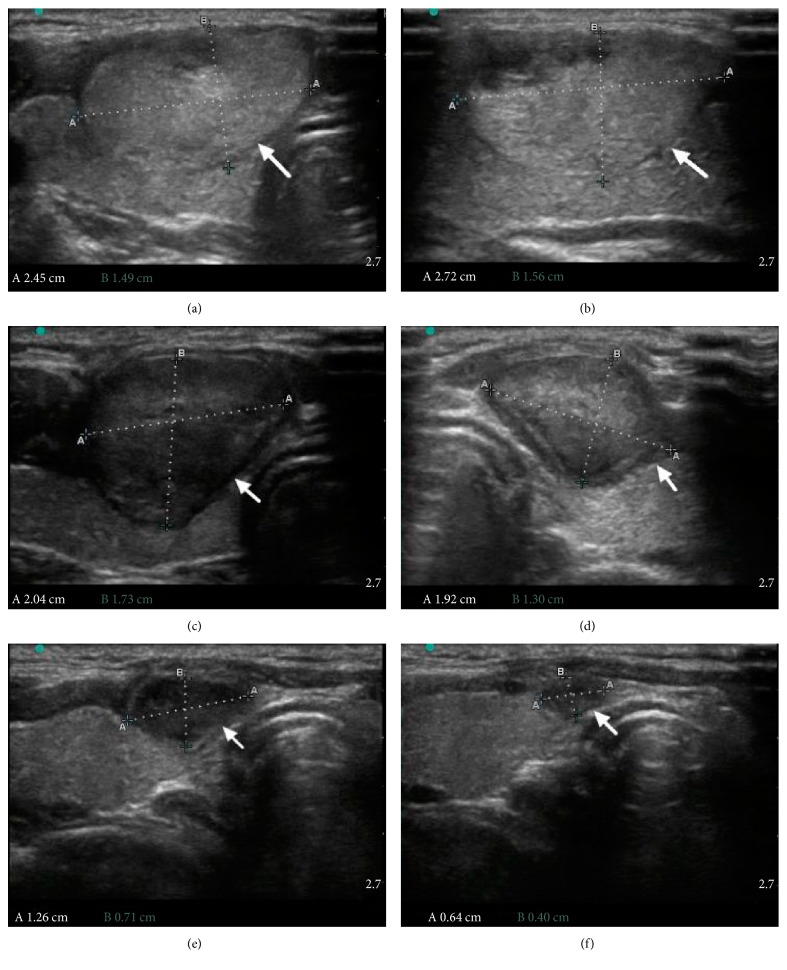
A 21-year-old female had a solid nodule in the left lobe of her thyroid gland. (a) The transverse of US examination showed a mainly solid nodule (arrow) which caused cosmetic problem in the superficial layer, and the volume of index nodule was approximately 5.40 ml (2.72 × 1.56 × 2.43 cm). (b) The long axis of index nodule. (c) At 1-month follow-up, US examination showed a little reduction in volume, and the volume was approximately 4.79 ml. (d) Three months after microwave ablation, the volume of the nodule significantly decreased to 3.75 ml. (e) Six months after the procedure, the index nodule shrunk to a volume of 0.96 ml. (f) At 1-year follow-up, the volume of the nodule greatly decreased to 0.08 ml, the VRR was 98.51%, and cosmetic grade significantly improved.

**Figure 2:**
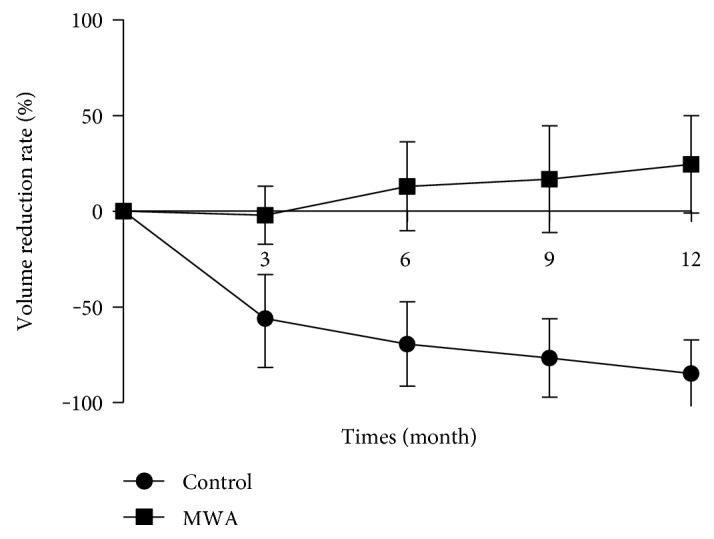
Comparison of the mean volume reduction rate (VRR) of the nodules at baseline (time of microwave ablation) and at follow-up after treatment. The VRR at 3-, 6-, 9-, and 12-month follow-ups was 55.98%, 69.31%, 76.65%, and 84.67% in the MWA group, respectively, while the VRR at 3-, 6-, 9-, and 12-month follow-up in the control group was 2.07%, −13.03%, −16.67%, and −24.53%, respectively.

After treatment, all nodules in the MWA group decreased in volume. The VRR in the MWA group was 30.53%, 55.98%, 69.31%, 76.65%, and 84.67% at the 1-, 3-, 6-,9-, and 12-month follow-ups, respectively. The largest diameter of thyroid nodules decreased from 2.92 ± 0.55 cm at baseline to 2.27 ± 0.99 cm, 1.82 ± 0.91 cm, 1.81 ± 0.59 cm, 1.58 ± 0.68 cm, and 1.38 ± 0.56 cm at 1-, 3-, 6-, 9-, and 12-month visits (*p* < 0.001) (Figures 1 and 2, Table 3). The nodules with VRR > 50% at 6- and 12-month follow-ups were 83.3% and 93.5%. On the other hand, the nodule volume in the control group got increased as time went by. The mean volume increased 24.53% at 12-month follow-up.

In the enrolled patients, with the diameter > 2 cm, though the nodule volume has greatly reduced after the procedure, no nodules showed complete disappearance at 12-month follow-up.

### 3.2. Improvement of the Clinical Symptoms and Cosmetic Problem

The symptom grading score was significantly reduced from 3.81 ± 1.99 to 0.96 ± 0.82 (*p* < 0.01) in the MWA group while it was stable in the control group. The cosmetic score was also improved in the MWA group from 2.81 ± 0.71 to 1.11 ± 0.31, (*p* < 0.01), while in the control group, the grade was unchanged when compared with baseline (Table 2).

### 3.3. Relation between VRR, Ablation Time (AT), and Baseline Thyroid Volume

**Figure 3:**
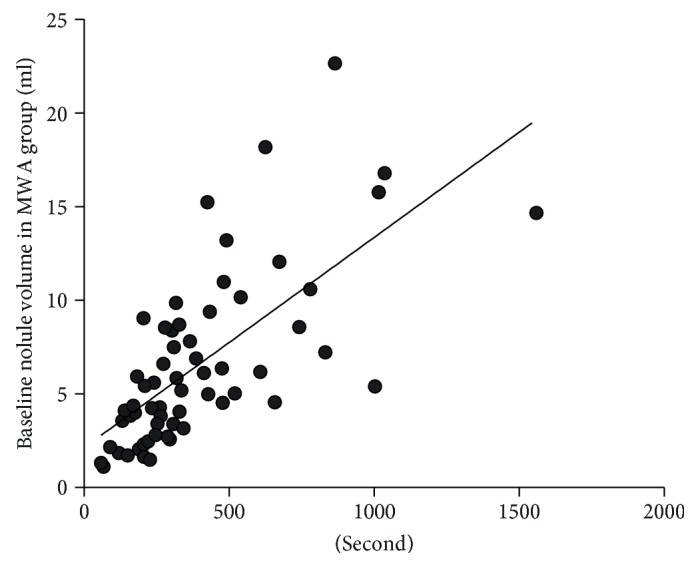
Positive correlation between ablation time and index nodule volume. *Y* = 0.011*x* + 2.12; *r*^2^ = 0.472; *p* < 0.001.

There was no correlation between VRR with the index volume. There was a linear positive correlation between the ablation time and baseline volume of the thyroid nodules, and the correlation coefficient was 0.68 (*p* < 0.001) (Figure 3).

### 3.4. Safety Profiles

All the patients who received microwave ablation are well tolerated to the procedure. Ten patients (13.3%) complained of various degrees of pain at the ablated site or pain radiating to the ear, shoulder, or teeth. The pain was totally relieved when the ablation was finished. No one needed analgesics. There was no hematoma formation developed. Ten patients (13.3%) that complained transient voice change due to lidocaine injection recovered within 24 hours after procedure. However, two patients (2.7%) that encountered voice change recovered within 2 months. Slight skin burn happened in one case (1.3%). One patient (1.3%) who suffered from Horner syndrome, mainly for ptosis and pupil shrinks, within 2 months recovered to normal. There were no serious complications such as esophageal perforation and tracheal injury.

## 4. Discussion

Thyroid nodules are very common disease. Most thyroid nodules are benign and asymptomatic, which do not need any treatment [17]. However, US-guided thermal ablation treatment may be considered for solid or mixed symptomatic benign nodules [18]. The value of current study is comparing the efficacy of the MWA with control groups. It demonstrated that MWA was an effective modality in decreasing volumes of benign thyroid nodules, as well as improving cosmetic grading scores and clinical symptoms. The comparison with the group of similar patients who were not treated indicated that the results were not due to spontaneous nodule change.

Microwave ablation is a minimally invasive technique to treat hepatic tumors that has been proven reliable, efficient, and safe [5, 19]. We conducted MWA for the treatment of benign thyroid nodules, and the efficacy is showed with a year of follow-up for these patients after the procedure. The nodule volume gradually reduced after MWA. According to the baseline, it can get nearly 85% of the volume reduction and greatly improve the clinical symptoms and cosmetic grading. Meanwhile, the side effects are mild. The thyroid nodules did not disappear after microwave ablation. However, it seemed to need a period of time for degradation by the immune system in the ablation area. The VRR at 3-, 6-, 9-, and 12-month follow-ups was 55.98%, 69.31%, 76.65%, and 84.67%, respectively. It proved to shrink in nodule volume significantly. Though microwave ablation will not lead to excessive destruction of thyroid, the thyroid hormones will not be affected [20].

The results among patients undergoing MWA in this study were superior to those among patients acting as control. It revealed that MWA significantly decreased TN volume in comparison with untreated patients who did experience TN size increase. The magnitude of volume reduction in this study is similar to studies conducted by RFA and other MWA studies [6, 8, 21], though there is no head-to-head studies which thermal modality is superior. Electromagnetic microwaves agitate water molecules in the surrounding tissue, producing friction and heat, thus inducing cellular death via coagulation necrosis. Microwave ablation of tumor center of the highest temperature can reach 100–120°C, not only can quickly kill tumor cells but also can solidify the surrounding blood vessels, and reduce the blood supply to the tissue [22].

Procedure-related major complications including recurrent laryngeal nerve paralysis and Horner syndrome occurred in two and one cases, respectively. Since the recurrent laryngeal nerve was close to the treated nodule when it was located in the danger triangle, we believe it was a technical error caused by directing the thermal energy too close to this region [23]. Horner syndrome, presenting as a combination of ptosis, miosis, and anhidrosis of the face, could be caused by thermal injury to the middle cervical sympathetic ganglion (mCSG) [24]. The mCSG is usually located at the lower level of thyroid gland, being lateral to the common carotid artery, and is visible in 41% of US images [25]. Under such circumstance, moving shot technique or incomplete ablation may be considered [19]. Ten patients (13.3%) complained of transient cervical pain related to the procedure. Ten patients (13.3%) encountered transient voice change due to lidocaine injection. Both were regarded as minor complications [26]. One patient experienced slight skin burn due to thermal effect as the nodule was very superficial. Some preventive measure was needed to be performed. The hydrodissection technique made by saline injection to the subcutaneous tissue was used, or cooling with the ice-box at the ablated site was applied.

This study has some limitations and shortcomings. Firstly, the major complication rate is a little higher than previous studies [20, 24, 27]. It seemed that the hydrodissection technique was not perfect. The injected fluid usually disappeared within some minutes; therefore, repeated fluid injection is necessary during the procedure. Moreover, lateral approach can increase recurrent laryngeal nerve injury when it is close to the nodules in the danger triangle. In addition, MWA has a much larger area of active heating compared with RFA [28]. Secondly, the enrolled nodules, with the largest diameter > 2 cm, have greatly reduced in nodule volume after the procedure; however, the treated nodules were smaller than 10 ml. Therefore, the volume reduction may be overestimated. No nodules showed complete disappearance at 12-month follow-up. It seemed that there is still not enough time for the degradation of the ablated tissues. Longer follow-up should be necessary for observation. Lastly, the study sample size is small and the study was conducted retrospectively.

In conclusion, MWA is an effective modality in decreasing volumes of benign thyroid nodules, as well as improving cosmetic grading and clinical symptoms, compared with the control group. However, prospective study with large-scale and long-term follow-up is necessary to be developed.

## Figures and Tables

**Table 1:** Demographic characteristics of the ablation group and control group. Normal ranges of the hormones are TSH 0.34~5.6 mIU/l, FT3 3.8~6.0 pmol/l, and FT4 7.86~14.41 pmol/l.

	Ablation group	Control group	*p* value
Cases (*n*)	75	40	—
Nodules (*n*)	90	54	—
Age (y)	39.38 ± 10.09	43.36 ± 9.88	0.187
Gender			
Male	11 (14.7)	11 (27.5)	—
Female	64 (85.3)	29 (72.5)	0.096
Ablation time (s)	418.2 ± 308.36	—	—
Power (w)	35.00	—	—
Location			
Left lobe (*n*)	36	23	—
Right lobe (*n*)	46	26	0.942
Isthmus (*n*)	8	5	—
TSH (mIU/l)	1.73 ± 1.88	1.34 ± 0.68	0.523
FT3 (pmol/l)	4.32 ± 0.55	4.30 ± 0.67	0.928
FT4 (pmol/l)	11.43 ± 4.30	10.12 ± 2.44	0.360

**Table 2:** Outcomes of the microwave ablation for thyroid nodules.

	Nodule volume (ml)						Cosmetic scores		Clinical symptoms	
	Baseline	1 month	3 months	6 months	9 months	12 months	Before	12 m (after)	Before	12 m (after)
Control	5.34 ± 3.88	—	5.18 ± 3.13	5.64 ± 3.86	6.35 ± 4.82	6.50 ± 4.36^a^	3.07 ± 0.61	2.92 ± 0.83	4.35 ± 1.27	4.07 ± 1.20
MWA	6.61 ± 4.65	4.03 ± 3.30	2.48 ± 2.27^b^	1.94 ± 1.73^b^	1.33 ± 1.45^b^	0.87 ± 0.99^b^	2.81 ± 0.71	1.11 ± 0.31^b^	3.81 ± 1.99	0.96 ± 0.82^b^
*p* value	0.229	—	0.001	<0.001	<0.001	<0.001	0.209	<0.001	0.206	<0.001

Mean volumes as mean ± standard deviation. ^a^*p* < 0.01, variate was compared with baseline in control group. ^b^*p* < 0.01, variate was compared with baseline in the MWA group.

**Table 3:** The changes in volume and diameter before MWA and at each follow-up.

	Baseline	1 month	3 months	6 months	9 months	12 months
Largest diameter (cm)	2.92 ± 0.55	2.27 ± 0.99^c^	1.82 ± 0.91^c^	1.81 ± 0.59^c^	1.58 ± 0.68^c^	1.38 ± 0.56^c^
Volume (ml)	6.61 ± 4.65	4.03 ± 3.30	2.48 ± 2.27^d^	1.94 ± 1.73^d^	1.33 ± 1.45^d^	0.87 ± 0.99^d^
VRR (%)	—	30.53 ± 38.94	55.98 ± 25.59	69.31 ± 22.03	76.65 ± 20.45	84.67 ± 17.37

Variates shown as mean ± standard deviation. ^c^*p* < 0.01, nodule largest diameter was compared with baseline in the MWA group. ^d^*p* < 0.01, nodule volume was compared with baseline volume in the MWA group.

## References

[1] Gharib H., Papini E. (2007). Thyroid nodules: clinical importance, assessment, and treatment. *Endocrinology and Metabolism Clinics of North America*.

[2] Chen Z., Xu W., Huang Y. (2013). Associations of noniodized salt and thyroid nodule among the Chinese population: a large cross-sectional study. *The American Journal Clinical Nutrition*.

[3] Guo H., Sun M., He W. (2014). The prevalence of thyroid nodules and its relationship with metabolic parameters in a Chinese community-based population aged over 40 years. *Endocrine*.

[4] Che Y., Jin S., Shi C. (2015). Treatment of benign thyroid nodules: comparison of surgery with radiofrequency ablation. *American Journal of Neuroradiology*.

[5] Yue W., Wang S., Wang B. (2013). Ultrasound guided percutaneous microwave ablation of benign thyroid nodules: safety and imaging follow-up in 222 patients. *European Journal of Radiology*.

[6] Li X. L., Xu H. X., Lu F. (2016). Treatment efficacy and safety of ultrasound-guided percutaneous bipolar radiofrequency ablation for benign thyroid nodules. *The British Journal of Radiology*.

[7] Papini E., Rago T., Gambelunghe G. (2014). Long-term efficacy of ultrasound-guided laser ablation for benign solid thyroid nodules. Results of a three-year multicenter prospective randomized trial. *The Journal of Clinical Endocrinology and Metabolism*.

[8] Jeong W. K., Baek J. H., Rhim H. (2008). Radiofrequency ablation of benign thyroid nodules: safety and imaging follow-up in 236 patients. *European Radiology*.

[9] Ha E. J., Baek J. H. (2014). Advances in nonsurgical treatment of benign thyroid nodules. *Future Oncology*.

[10] Pacella C. M., Mauri G., Achille G. (2015). Outcomes and risk factors for complications of laser ablation for thyroid nodules: a multicenter study on 1531 patients. *The Journal of Clinical Endocrinology and Metabolism*.

[11] Park H. S., Baek J. H., Park A. W. (2017). Values and limitations of the comparing thyroid radiofrequency and microwave ablation using propensity score. *Endocrine*.

[12] Mader O. M., Tanha N. F., Mader A., Happel C., Korkusuz Y., Grünwald F. (2017). Comparative study evaluating the efficiency of cooled and uncooled single-treatment MWA in thyroid nodules after a 3-month follow up. *European Journal of Radiology Open*.

[13] Na D. G., Lee J. H., Jung S. L. (2012). Radiofrequency ablation of benign thyroid nodules and recurrent thyroid cancers: consensus statement and recommendations. *Korean Journal of Radiology*.

[14] Cibas E. S., Ali S. Z. (2009). The Bethesda system for reporting thyroid cytopathology. *Thyroid*.

[15] Park H. S., Baek J. H., Park A. W., Chung S. R., Choi Y. J., Lee J. H. (2017). Thyroid radiofrequency ablation: updates on innovative devices and techniques. *Korean Journal of Radiology*.

[16] Baek J. H., Kim Y. S., Lee D., Huh J. Y., Lee J. H. (2010). Benign predominantly solid thyroid nodules: prospective study of efficacy of sonographically guided radiofrequency ablation versus control condition. *American Journal of Roentgenology*.

[17] Durante C., Costante G., Lucisano G. (2015). The natural history of benign thyroid nodules. *JAMA*.

[18] Gharib H., Papini E., Garber J. R. (2016). American Association of Clinical Endocrinologists, American College of Endocrinology, and Associazione Medici Endocrinologi medical guidelines for clinical practice for the diagnosis and management of thyroid nodules - 2016 update. *Endocrine Practice*.

[19] Feng B., Liang P., Cheng Z. (2012). Ultrasound-guided percutaneous microwave ablation of benign thyroid nodules: experimental and clinical studies. *European Journal of Endocrinology*.

[20] Heck K., Happel C., Grünwald F., Korkusuz H. (2015). Percutaneous microwave ablation of thyroid nodules: effects on thyroid function and antibodies. *International Journal of Hyperthermia*.

[21] Yue W. W., Wang S. R., Lu F. (2017). Radiofrequency ablation vs. microwave ablation for patients with benign thyroid nodules: a propensity score matching study. *Endocrine*.

[22] Simon C. J., Dupuy D. E., Mayo-Smith W. W. (2005). Microwave ablation: principles and applications. *Radiographics*.

[23] Lang B. H., Woo Y. C., Wong I. Y., Chiu K. W. (2017). Single-session high-intensity focused ultrasound treatment for persistent or relapsed graves disease: preliminary experience in a prospective study. *Radiology*.

[24] Kim C., Lee J. H., Choi Y. J., Kim W. B., Sung T. Y., Baek J. H. (2017). Complications encountered in ultrasonography-guided radiofrequency ablation of benign thyroid nodules and recurrent thyroid cancers. *European Radiology*.

[25] Shin J. E., Baek J. H., Ha E. J., Choi Y. J., Choi W. J., Lee J. H. (2015). Ultrasound features of middle cervical sympathetic ganglion. *The Clinical Journal of Pain*.

[26] Sacks D., McClenny T. E., Cardella J. F., Lewis C. A. (2003). Society of Interventional Radiology clinical practice guidelines. *Journal of Vascular and Interventional Radiology*.

[27] Baek J. H., Lee J. H., Sung J. Y. (2012). Complications encountered in the treatment of benign thyroid nodules with US-guided radiofrequency ablation: a multicenter study. *Radiology*.

[28] Liang P., Dong B., Yu X. (2001). Computer-aided dynamic simulation of microwave-induced thermal distribution in coagulation of liver cancer. *IEEE transactions on Bio-medical Engineering*.

